# Checklist of coral reef fishes of Darvel Bay, Sabah, Malaysian Coral Triangle, with a note on the biodiversity and community structure

**DOI:** 10.3897/BDJ.10.e79201

**Published:** 2022-08-12

**Authors:** Nur Farhana-Azmi, B. Mabel Manjaji-Matsumoto, Nasrulhakim Maidin, Jonathan Balang John, Elvin Michael Bavoh, Ejria Saleh

**Affiliations:** 1 Borneo Marine Research Institute, Universiti Malaysia Sabah, 89400 Kota Kinabalu, Sabah, Malaysia Borneo Marine Research Institute, Universiti Malaysia Sabah 89400 Kota Kinabalu, Sabah Malaysia; 2 Sabah Parks, P.O. Box 10626, 88806 Kota Kinabalu, Sabah, Malaysia Sabah Parks, P.O. Box 10626 88806 Kota Kinabalu, Sabah Malaysia

**Keywords:** anthropogenic impacts, ichthyology, land-use change, Malaysian Borneo, overfishing

## Abstract

The Darvel Bay is a large semi-enclosed bay with spectacular natural land and seascape. The inward side of the Bay has only been recently known to be an important foraging ground for the endangered, threatened and protected (ETP) elasmobranch species, such as the Whale Shark and mobulid rays. Following a recent scientific expedition, we present a checklist of the coral reef fishes of Darvel Bay. A note on the biodiversity and community structure is presented, based on our analysis using diversity indices, univariate and multivariate approaches. Seven natural coral reefs comprising two fringing reefs and five patch reefs, were surveyed at 10 m depth using underwater visual census (UVC) and baited remote underwater video station (BRUVS) methods. A diverse list of 66 species of reef fishes from 17 families is recorded. However, this is overwhelmingly dominated by the small-sized omnivorous damselfish, family Pomacentridae (62%; N = 1485 individuals). Species richness and abundance were observed to increase at sites surveyed furthest from the coast within the Bay. Significantly distinct reef fish assemblages were observed between three priori groups, based on proximity to shore (ANOSIM, R = 0.65, p < 0.05). SIMPER analysis further revealed that 22 species of the total reef fish species recorded drive 76% dissimilarities between the groups. The pattern of the reef fish communities observed, reflected as a logseries distribution model, is that commonly found in disturbed habitats or habitats characterised by restricted resources in a community, where the dominant species takes up a high proportion of available resources. The ecological indices (Shannon-Wiener Diversity Index, 2.05; Simpson Index of Diversity, 0.79; Simpson Dominance Index, 0.20; and Pielou’s Evenness Index, 0.43), all reflect the relatively low diversity and uneven species distribution of the reef fish community. We conclude that the present status of the coral reef fish community dominating Darvel Bay as having undergone a rapid shift in structure following intense and rampant fishing pressure, as reported by the media.

## Introduction

Darvel Bay, the largest bay located in the east coast of Sabah, is interconnected with the Pacific Ocean through the Sulu Sea (Ditlev et al. 1999). It is composed of mainly fringing reefs surrounding its islands and small, scattered patch reefs that form coral chains into the Philippine Sulu Archipelago (Ditlev 2003). The reefs are sheltered and characterised by the turbid waters with high humic content, especially towards the inner part of the Bay (Ditlev 2003) with water circulation pattern mainly influenced by tidal currents (Ditlev 2003, Saleh et al. 2007). Apart from coral reefs, stretches of seagrass, mangroves and seaweed beds could be observed along the coast and the surrounding islands (Norhadi 1996, Pilcher 1996, De Silva et al. 1999).

Located at the north-corner of the Coral Triangle region, Darvel Bay is recognised as one of the most biologically diverse marine environments in the world (Ditlev et al. 1999, Waheed and Hoeksema 2012); however, its marine biodiversity has remained under-studied (Wood et al. 2004, Ho and Kassem 2009, Affendi et al. 2012). Coral species diversity of Semporna and Darvel Bay is incredibly high, with multiple coral species found to be endemic to north-eastern Sabah (Waheed and Hoeksema 2012), which is not found elsewhere. The highest number of mushroom coral species are also recorded here, surpassing other areas in the Coral Triangle region (Hoeksema et al. 2004, Hoeksema 2007, Hoeksema 2008), signifying its rich biodiversity. Literature pertaining to Darvel Bay’s fish species diversity and composition, however, remains very limited. A previous expedition back in 1998 had reported the status of marine resources and fisheries activities in Darvel Bay, highlighting fish species found at Lahad Datu landing sites, caught within the Bay through commercial and artisanal means (De Silva et al. 1999). Since then, apart from a few Reef Check surveys and a single publication by Pilcher and Cabanban (2000), data pertaining to reef fish composition of Darvel Bay remained scarce.

According to past literature, the biodiversity of Darvel Bay is under threat from the increasing pressure of coastal development activities and destruction of marine ecosystems (Cem and Assim 1996, De Silva et al. 1999). Coastal development along the Bay is often carried out without any detailed study of the dynamic coastal processes, including coastal erosion, sedimentation and depletion of water quality (Othman and Lee 1999). Destructive fishing practices, such as blast fishing, muro ami (type of fishing technique that involves encircling a large net on top of a coral reef and skin divers pounding the net to drives fishes out from the reef into the net) and poison fishing are also rampant in the region (Pilcher and Cabanban 2000). On average, 80% of the area sampled were below 30% live coral cover, noting high disturbance indicators, such as blast impact, discarded fishing nets and trash across the reef (Reef Check Malaysia, 2013, Reef Check Malaysia 2017, Reef Check Malaysia, 2019). The same report also highlighted low abundance of indicator fish species, suggesting the presence of high fishing pressure. Sheltered locations within the Bay garnered economic attention as it held great potential for aquaculture activities involving mainly seaweed farming as well as fish and oyster floating farms (Saleh et al. 2007), thereby compounding associated anthropogenic impacts in the Bay. An area of 70,000 hectares within the Bay, located between Semporna and Lahad Datu Districts, have been established as an Aquaculture Industrial Zone (Saleh et al. 2007) with most current aquaculture sites concentrated in the coastal area close to the Kunak District.

A recent study highlights the conservation importance of the Bay as a critical habitat for the endangered whale shark (*Rhincodontypus*, Smith 1828) (Araujo et al. 2019). Satellite telemetry evidence signifies the potential of Darvel Bay as part of the migratory corridor connecting to Palawan, Phillipines through a coral chain (Araujo et al. 2019). Apart from that, anecdotal evidence also suggests occasional co-occurrence of whale shark with other filter-feeding megafauna, including *Mobulabirostris* Walbaum 1792, *M.kuhlii* Valenciennes 1841, *M.mobular* Bonnaterre 1788 and *Balaenopteraedeni* Anderson 1879 that highlights the potential high prey density in the area. Interestingly, this recent discovery coincides with high chlorophyll-α productivity in the Bay, between November and March (Walsh 2009, Gordon et al. 2011), which may be prompting the whale shark long-distance migration (Araujo et al. 2019). Increased conservation importance, as well as acknowledging the rising threats towards the biodiversity of the Bay, has recently been proposed as a de-facto marine protected area (MPA) (Bernama 2019a, Ralon 2019, Bernama 2021a). This effort set by the State Government of Sabah also aligns with the objective set by the Coral Triangle Initiative and Sulu-Sulawesi Seascape Project that sets out to establish and manage protected area networks, particularly the migratory corridor for threatened and endangered species.

Following this plan, Darvel Bay Scientific Expedition was conducted on 11 – 15 February 2019, aiming to assess the status of its biodiversity and to provide preliminary baseline data of Darvel Bay as one of the proposed areas for such gazettement. Coral reef fishes are undeniably a crucial biological component of the marine ecosystem and ecological measures of species abundance and diversity could provide hindsight to the health status of the reefs. In this survey, we aim to provide the recent baseline information on the diversity, abundance and community structure of reef fish in the area.

## Methodology

### Study area

**Figure 1.**
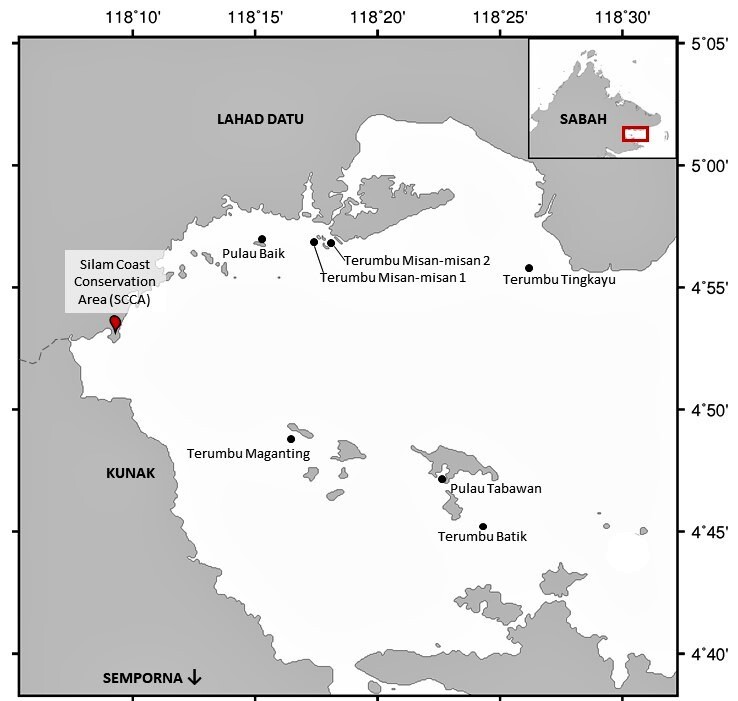
Map of Darvel Bay. Black dots indicate sampling sites.

Administratively located within Tawau jurisdiction, Darvel Bay borders three main districts of Sabah - with Lahad Datu on the north side, Kunak in the middle and Semporna on the south of the Bay. A field survey was conducted on 11 – 15 February 2019 at seven reefs (Fig. 1) comprised of two fringing reefs; Pulau Baik and Pulau Tabawan [Note: Pulau is ‘island’ in Bahasa Malaysia] and five patch reefs; Terumbu Misan-misan 1, Terumbu Misan-misan 2, Terumbu Maganting, Terumbu Batik and Terumbu Tingkayu [Note: Terumbu is ‘reef’ in Bahasa Malaysia].

### Field sampling

Surveys were conducted at 10 m depth at all sites during daylight hours 0900 to 1500 following underwater visual census (UVC), coupled with baited remote underwater video stations (BRUVS). UVC was carried out over a 100 m transect where the diver recorded fish species and abundance data every 3 m distance ahead of them within 3 m transect width and 3 m height from the substrate. Mobile species were recorded first and were followed by those that were less mobile. BRUVS were deployed simultaneously at least 100 m away from the transect and were left for 60 minutes recording time before retrieving. For every deployment, 0.5 kg of chopped scads (*Decapterus* spp.) was used as a standard bait for this research as scads are known to have high consistency of fish oil that will increase bait plume to attract more fish (Dorman et al. 2012, Harvey et al. 2013, Wraith et al. 2013) and increase samples recorded. Footages were recorded using an action camera attached to the BRUVS unit, one metre away from the bait. It is also worth noting that, due to time constraint, each site was sampled only once, without replicates. Fish species recorded were identified to species level using Eschmeyer's Catalog of Fishes (Fricke et al. 2022).

### Data treatment and statistical analysis

Fish abundance data were combined both from UVC and BRUVS and average abundances were analysed using univariate and multivariate methods. General ecological measures were calculated including species diversity (Shannon-Weiner Index and Simpson Diversity Index), Dominance Index and Pielou’s Evenness Index were determined using Paleontological Statistics (PAST Inc. Palaeontological Association) (Hammer et al. 2001). The species accumulation curve was constructed by using the EstimateS V.9 (EstimateS: Biodiversity Estimation Software) (Colwell and Elsensohn 2014) with non-parametric, abundance-based estimators Chao1 being used to estimate species richness. Magurran (2004) highlighted that, amongst the abundance-based species richness estimators, Chao1 depends on the rare species; singletons (species with one individual) and doubletons (species with two individuals) to estimate species richness and has been used in many studies to reliably estimate marine fish species richness (Foggo et al. 2003). The species accumulation curves show sampling adequacy by illustrating the rate at which additional species are found with increased sampling effort, resulting in the curve arch upwards. Meanwhile, when the curve flattens and reach an asymptote, it is unlikely additional species will be discovered with increased sampling efforts (Magurran 2004).

Multivariate analysis was carried out using PRIMER-e V.7 (PRIMER-E: Plymouth, United Kingdom) with PERMANOVA package add-ons (Clark and Gorley 2015). Abundance data were square-root transformed to weigh down the influence of some highly abundant species and analysis was carried out using the Bray-Curtis dissimilarity matrix. The non-metric multi-dimensional scaling (nMDS) and classic hierarchal cluster analysis was conducted to represent the groupings, based on reef fish composition at each site. Similarity profile (SIMPROF) analysis was then conducted to validate output dendogram and contours were applied to the nMDS plot to show significant groupings of the sites (Clarke et al. 2008). Analysis of Similarity (ANOSIM) was carried out to test for differences between/amongst the priori groups classified, based on proximity to shore and reef type (Table 1). Similarity percentage analysis (SIMPER) was performed to observe the average dissimilarity percentage between the groups, as well as the contributary species that drive the differences in assemblage observed between sites.

## Result

**Figure 2.**
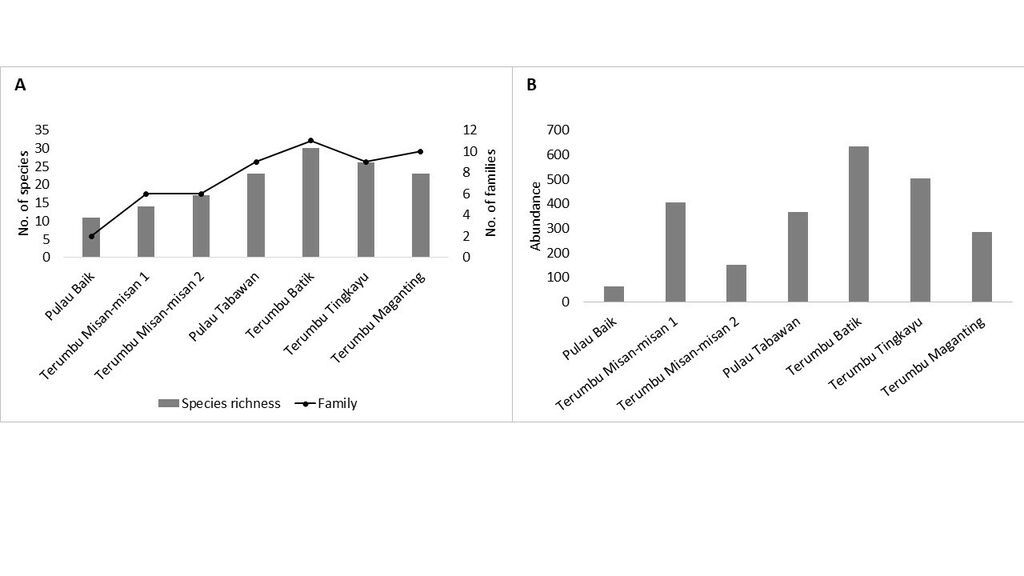
A) Total number of species and families. B) Total abundance at seven sites surveyed in Darvel Bay.

A total of 2402 individuals of reef fishes from 66 species and 17 families were identified throughout the survey (Table 2). Grey Demoiselle, *Chrysipteraglauca*, was the most abundant species overall, constituting 16.99% of the community, followed by Yellow-spotted Chromis, *Chromisnotata*, at 13.78% and schooling Striped-eel Catfish, *Plotosuslineatus*, at 13.74%. Fish assemblage recorded reflected a typical reef fish composition mainly dominated by damselfish (Pomacentridae), which constituents for 61.82% (N = 1485 individuals) from 17 genera. Broken down to each survey site, Terumbu Batik and Terumbu Tingkayu are the two most diverse sites with 30 species (11 families) and 26 species (9 families) recorded correspondingly (Fig. 2). These two sites also had the highest number of abundances recorded, contributing to 47.25% of the overall assemblage observed (Fig. 2). Pulau Baik had the lowest species richness and abundance with only 66 individuals recorded from 11 species recorded; mainly comprised of small-sized damselfish and wrasse.

**Figure 3.**
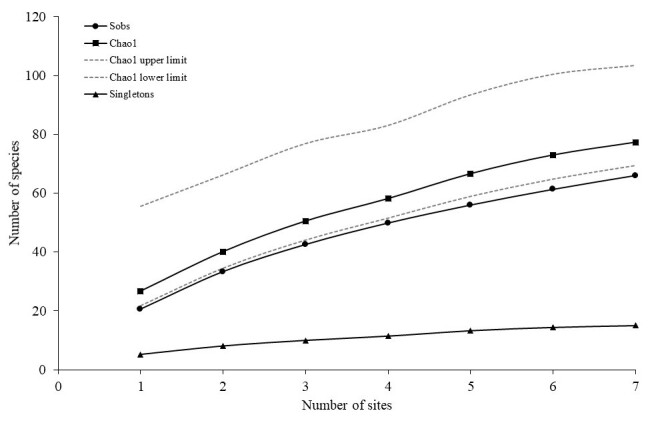
Observed species accumulation curve (Sobs) with Chao1 estimator and singletons.

Observed species accumulation curve (Sobs) reached maxima without indication of reaching asymptotic ends (Fig. 3). The extrapolation of species richness estimators Chao1 resulted in a higher curve than observed data (Sobs). The Chao1 estimator suggested a total of 77.25 reef fish species compared to only 66 species observed, resulting between 4.88% to 36.16% of estimated proportion of unsampled species (Fig. 3), yet to be documented. The Chao1 estimator reliability depends on adequate sample size, which means the proportion of singletons should be less than 50% (Chao 1987). The proportion of singletons recorded in this study at 22.73% suggests sufficient sample size recorded to validate Chao1 as a reliable species richness estimator.

**Figure 4.**
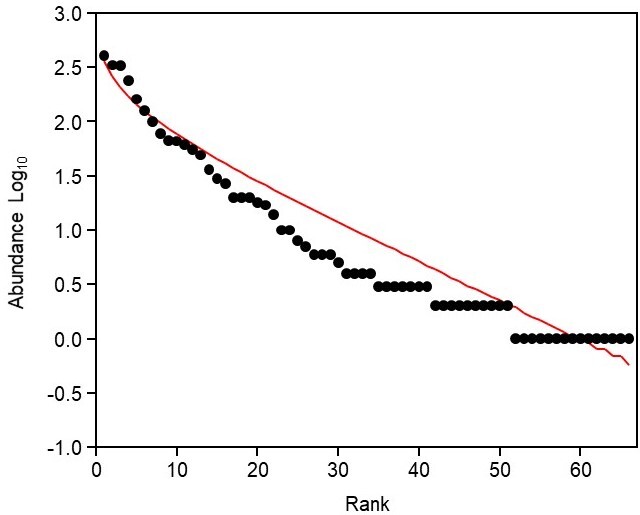
Rank abundance curve (RAC) showing reef fish community fits logseries model.

The Rank abundance curve revealed fish community structure following logseries model (α = 12.55, X^2^ = 0.99, p <0.05) (Fig. 4). Overall, ecological indices show relatively fair value, in which the Shannon-Wiener Diversity Index, H’ = 2.05, Simpson Diversity Index, 1-D = 0.79, Simpson Dominance Index, D = 0.20 and Pielou’s Evenness Index, E = 0.43. Ecological indices at each site are presented in Table 3.

**Figure 5.**
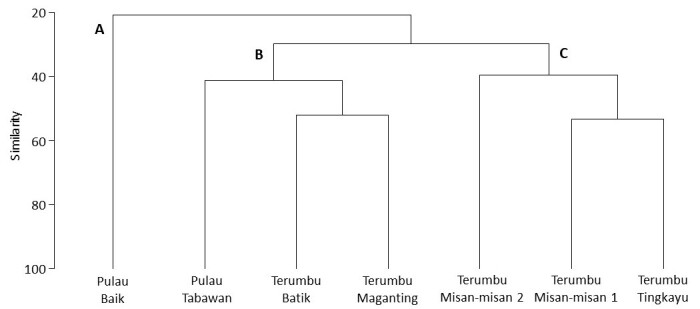
Hierarchical cluster analysis revealed two main clusters and a single outlier, based on a different reef fish assemblage at each site.

**Figure 6.**
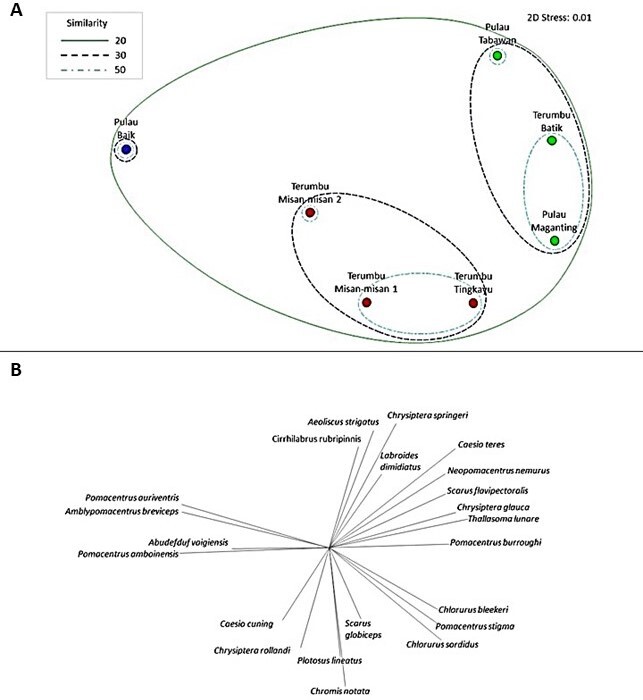
A) Non-metric multidimensional scaling (nMDS) ordination with SIMPROF contours at 20, 30 and 50 similarity slices. Priori groups between sites surveyed in which Blue indicate single outlier A, green indicate Cluster B and red indicate Cluster C. B) Vectors of 22 species identified in SIMPER analysis.

The dendogram (hierarchical cluster analysis), based on reef fish assemblages across sites, reflects the proximity to the Bay (Cophenetic coefficient: 0.85). SIMPROF grouped the sites into two distinct clusters with one single outlier. Outlier A is represented by Pulau Baik, cluster B comprises of Terumbu Maganting, Pulau Tabawan and Terumbu Batik and cluster C comprises of Terumbu Misan-misan 1, Terumbu Misan-misan 2 and Terumbu Tingkayu (Fig. 5) indicating significant differences in fish assemblages. At 30% similarity, nMDS ordination shows two main groups with one single outlier (Fig. 6), representing different proximity to shore as a potential factor that drives difference fish assemblages. At 50% similarity, smaller subset clusters was observed to form within both cluster B and C. Fish assemblage across clusters were significantly different (ANOSIM, R = 0.65, p < 0.05), based on proximity to shore; however, it was not significant for reef type (ANOSIM, R = 0.11, p > 0.05) (Table 4). SIMPER analysis revealed the average percentage of dissimilarities between the priori groups were 76.01%, contributed by 22 species of reef fishes (Table 5, Fig. 6).

Pulau Baik was characterised by mostly damselfish species, *Pomacentrusamboinensis*, *P.auriventris*, *Abudefdufvaigiensis* and *Amblypomacentrusbreviceps*. Cluster B shared average similarity of 44.89%, contributed by common species *Chrysipteraglauca*, *P.burroughi*, *Caesioteres*, *Thallasomalunare* and *Scarusflavipectoralis*, whereas Cluster C with average similarity of 44.21% were characterised by *Chromisnotata*, *Plotosuslineatus*, *Caesiocuning* and *Scarusglobiceps*.

## Discussion

Reef fish assemblage recorded reflects a typical reef fish community, dominated mainly by small-sized damselfishes (Pomacentridae) and wrasse (Labridae), which are not unusual as both groups are highly diverse and naturally occurring in large numbers on tropical coral reefs (Sale 1993). These groups are not usually targeted species in fisheries and were not preferred by artisanal fishermen for consumption; hence, this might explain its prevalence on the reef. The scarcity for reef fish families commonly important to fisheries, such as groupers (Epinephelidae), snappers (Lutjanidae), jacks (Carangidae), emperors (Lethrinidae) and sweetlips (Haemulidae), as well as schooling planktivorous fish, such as caesionids (Lutjanidae) and commercial small-sized forage fishes (Scombridae) in this study, may be an indication of overfishing in the region (Stallings 2009, Longenecker et al. 2011). It is also important to note this time-sensitive expedition renders limited surveys to be taken place. Since the observed species accumulation curve (Fig. 3) did not reach the asymptotic ends, increased sampling effort is expected to discover more species (Chao 1984). Larger mesopredators of interest tend to be elusive and had been reported to shy away from divers, thus may be contributing to their low abundance recorded during a typical underwater visual census survey (Harvey et al. 2013). However, the scarcity of top predators and mesopredators in the region is likely, considering very low abundance of these species was recorded despite the use of bait via BRUVS, that negates the presence of divers. This result is consistent with Pilcher and Cabanban (2000) who conducted a UVC survey method in the region in 1998, which reported 71 species of reef fishes from 19 families were recorded, noting low abundance of commercial species. The same study also reported low abundance and diversity of caesionids with its presence being noted as higher in a healthy reef while being totally absent at degraded reef in the region.

The low abundance of commercial species and predatory fishes in the region is plausible due to Darvel Bay's historical and ongoing fishing pressure. Destructive fishing methods, such as blast fishing and poison fishing (Pilcher and Cabanban 2000, Biusing 2001, Burke et al. 2002) may have suppressed the recovery of targeted fish populations. An area known for mass aggregation of groupers and rabbitfishes in the southeast of Darvel Bay had been devastated by fish bombing activities in the 1980s, resulting in a rapid decline of catches (Daw 2004). According to media, illegal, unreported and unregulated (IUU) fishing in Sabah causes an estimate of RM6 billion loss every year, highlighting that only 50% of the fish caught in national waters make their way to the local market, while the rest remains untraceable (Bernama 2019b). This suggests that destructive fishing practices in Darvel Bay and its proximate waters are still ongoing despite increased enforcement, with recent arrests made into national headlines (Bernama 2019c, Toyos 2019, Bernama 2021b, Miwil 2021). Although illegal, arrests made on fish bombing activities remain relatively low as the authorities are mostly hampered by limited information on the location and timing of the bombing occurrence (Wood and Ng 2016), thus hindering effective arrest across large areas of marine waters. Live reef fish trade (LRFT) activities had been reported to be very active in the region, consisting of large holding operations, which transport 12-17 tonnes of live fish, commonly groupers to Hong Kong (Daw 2004). This further incentivises the use of poison by fishers to target highly-prized species, such as *Plectropomus* spp. and *Epinephelus* spp. Northern and eastern parts of Darvel Bay were subjected to commercial trawling activities (Biusing 2001), although further offshore, these may be aggravating the existing impact of overfishing and habitat destruction within the Bay.

Reef fish assemblage differed significantly based on their proximity to shore. This suggests ongoing anthropogenic factors that are tied to "distance from shore" as a proxy, may be influencing different assemblages observed. The inner bay located near the mainland is subjected to high sedimentation, resulting in very turbid reefs (Waheed and Hoeksema 2012, Farhana-Azmi 2019). Coastal zones of Darvel Bay receive freshwater influx from the main Segama River as well as being surrounded by alluvial plains and tidal swamps. This acts as numerous water catchment areas, thus contributing to the high sedimentation of the inner bay closest to the mainland (Cem and Assim 1996, Saleh et al. 2007, Santodomingo et al. 2021). Apart from that, coastal sedimentation was further intensified by numerous development projects taking place in the Silam Coast of Darvel Bay (De Silva et al. 1999). The coastal area and its scattered islands of Darvel Bay also experience land-use changes due rampant deforestation and conversion of the islands into palm oil plantations (Saleh et al. 2007). This had been reported to contribute to high organic load, nutrients and toxic substances in nearshore areas of Darvel Bay (Saleh et al. 2007). Reefs of Pulau Baik were observed to be very turbid (Farhana-Azmi 2019) compared to the other reefs surveyed, hence may explain its distinct reef fish assemblage, mainly dominated by damselfishes. BRUVS footage of the area revealed a relatively turbid reef with visually high nutrient indicator algae coverings. Due to its proximity to the coastline and a nearby fish farm (Santodomingo et al. 2021), the reefs of Pulau Baik may be negatively impacted by sedimentation as well as experiencing a high organic influx. This may facilitate algae growth, which are favoured by opportunistic damselfish and small labrids, while reducing other piscivorous species (Wilson et al. 2010). High abundance of herbivorous-detritivorous damselfishes, such as *Pomacentrusambonensis* and *Abudefdufvaigiensis* are common inhabitants of anthropogenically-affected reefs (Khalaf and Kochzius 2002). Although they remain relatively closer to the Bay’s estuary, Terumbu Misan-misan 1, Terumbu Misan-misan 2 and Terumbu Tingkayu did not suffer heavy sedimentation as compared to Pulau Baik (Farhana-Azmi 2019).

Sites located further offshore from the inner bay were observed to have higher abundance and fish species richness, potentially due to visibly improved reef conditions. Low sedimentation and clear visibility were observed throughout the survey at sites located further away from the inner bay. Sites located furthest from coastline namely Pulau Tabawan, Terumbu Batik and Terumbu Maganting contributed to the high abundance of parrotfishes. In Sabah, parrotfish are high on the menu, often targeted by artisanal fisherman (Lee and Chou 2003). However, high abundance of parrotfishes observed at these sites suggest these areas may be experiencing lower fishing pressure potentially due to their relatively further distance from the coast, hence reducing fishers’ accessibility. Absence of sensitive parrotfish species, such as *Bolbometoponmuricatum* (Valenciennes 1840) and *Chlorurus* spp., had been documented with increasing human densities and reefs being open to fishing (Bellwood et al. 2011). Although we did not observe *B.muricatum* per se, relatively high abundance of *Chlorurus* spp. alongside other parrotfish species recorded at Cluster C may indicate lower fishing pressure at sites located furthest from inner bay. The increased distance between a reef and human settlement (shore) had been associated with reduced fishers accessibility to the site, consequently reducing fishing pressure and other associated anthropogenic impacts (Nyström et al. 2000, Andersson 2002, Advani et al. 2015). In a recent study by Santodomingo et al. (2021), the density of marine-based litter, such as fishing nets/lines, were found to be higher on reef localities closer to the inner bay, constituting up to 55% of marine litter in Misan-misan reef. This indicates higher fishing pressure at the inner bay as compared to localities nearer to the centre of the Bay (Santodomingo et al. 2021); thus, elucidating fishing pressure may be one of the proxies separating the sites (Cluster B and C), based on proximity to shore.

Darvel Bay's historical and ongoing IUU fishing activities and rapid land-use change may cause a possible phase shift in macroalgae communities resulting in reef fish community observed to follow a logseries model. The logseries model is commonly used to characterise the biological community experiencing disturbances and/or living in restricted environmental conditions (Cielo Filho et al. 2002, Hill and Hamer 2004). The logseries model present a pattern of monopoly by a few dominant species and uneven distribution of species in a community (Cielo Filho et al. 2002; Magurran 2004). Chronic anthropogenic activities had been reported to cause shifts in the benthic community structure of stony corals to macroalgae-dominated landscape, by limiting the formation of complex reef habitat, which supports diverse organisms (Jones et al. 2004; Graham et al. 2006; Renfro and Chadwick 2017). This consequently reduces fish diversity in the affected area and leads to dominance of opportunistic species (Whittaker 2019). In this study, a pattern of dominance is exhibited by a few damselfish species and communities exhibit uneven species distribution across all sites, particularly at sites closest to the inner bay.

The results, presented here, serve as preliminary fish species recorded in Darvel Bay in recent years. In comparison with a similar survey conducted by Pilcher and Cabanban (2000), this survey reports almost similar findings. Consistencies in terms of the presence of high dominance of small-sized damselfish and wrasse, as well as scarce recordings of commercially-important species, may suggest persistent fishing pressure in the region is active. As such, we point out IUU fishing, overfishing as well as land-use pollution, are likely the primary reasons behind the absence of larger mesopredators, which are commercially-important fish species. Further investigations and long-term monitoring of the observed pattern of reef fishes are recommended. Close monitoring of reef fish assemblages, including recordings of other metadata, such as size, biomass and associated feeding guild, may illustrate a better understanding of the dynamics of reef fish community and serve as a bioindicator of habitat degradation or increased anthropogenic impacts. Gazettement of Darvel Bay as an MPA is expected to improve the fish abundance and diversity in the area with sustainable marine resource management as well as diligent enforcement by authorities against destructive fishing practices. Community-based resource management can be employed by MPA management considering the closely-linked livelihood of many coastal communities residing in Darvel Bay as they will be one of the major stakeholders in establishing Darvel Bay as an MPA.

## Figures and Tables

**Table 1.** Site, with their proximity to shore of the Bay; near or far and reef type; fringing or patch

**Site**	**Proximity to shore**	**Reef type**
Pulau Baik	Near	Fringing
Terumbu Misan-misan 1	Near	Patch
Terumbu Misan-misan 2	Near	Patch
Pulau Tabawan	Far	Fringing
Terumbu Batik	Far	Patch
Terumbu Tingkayu	Near	Patch
Terumbu Maganting	Far	Patch

**Table 2.** Reef fish species list at each reef surveyed. Mean is the mean number of the individuals between locations

Species name	N	Mean ± S.E	Pulau Baik	Terumbu Misan-misan 1	Terumbu Misan-misan 2	Pulau Tabawan	Terumbu Batik	Terumbu Tingkayu	Terumbu Maganting
Apogonidae									
*Cheilodipterusartus* (Smith 1961)	1	0.14 ± 0.14	-	-	-	-	-	-	1
Carangidae									
*Pseudocaranxdentex* (Bloch & Schneider 1801)	1	0.14 ± 0.14	-	-	-	-	-	-	1
Centriscidae									
*Aeoliscusstrigatus* (Günther 1861)	30	4.28 ± 4.28	-	-	-	30	-	-	-
Chaetodontidae									
*Chaetodonoctofasciatus* (Bloch 1787)	6	0.85 ± 0.59	-	-	-	-	2	4	-
*Chelmonrostratus* (Linnaeus 1758)	2	0.28 ± 0.28	-	-	-	-	2	-	-
Ephippidae									
*Plataxpinnatus* (Linnaeus 1758)	1	0.14 ± 0.14	-	-	-	-	1	-	-
Epinephelidae									
*Anyperodonleucogrammicus* (Valenciennes 1828)	1	0.14 ± 0.14	-	-	-	-	-	-	1
*Cephalopholisargus* (Schneider 1801)	14	2 ± 0.43	-	3	2	1	3	2	3
*Epinephelusmerra* (Bloch 1793)	1	0.14 ± 0.14	-	-	-	-	-	-	1
*Epinephelusongus* (Bloch 1790)	3	0.42 ± 0.42	-	-	-	-	-	-	3
Labridae									
*Anampsesmeleagrides* (Valenciennes 1840)	10	1.42 ± 1.42	-	10	-	-	-	-	-
*Bodianusmesothorax* (Bloch & Schneider 1801)	4	0.57 ± 0.2	-	-	-	1	1	1	1
*Cheilinusfasciatus* (Bloch 1791)	17	2.42 ± 0.89	2	5	-	6	3	1	-
*Choerodonanchorago* (Bloch 1791)	1	0.14 ± 0.14	-	-	-	-	-	-	1
*Cirrhrilabrusrubripinnis* (Randall & Carpenter 1980)	20	2.85 ± 2.85	-	-	-	20	-	-	-
*Corisaurilineata* (Randall & Kuiter 1982)	3	0.42 ± 0.29	-	1	-	-	-	2	-
*Diproctacanthusxanthurus* (Bleeker 1856)	1	0.14 ± 0.14	-	-	-	1	-	-	-
*Halichoereschrysus* (Randall 1981)	1	0.14 ± 0.14	-	-	-	-	-	-	1
*Halichoereshortulatus* (Lacepède 1801)	1	0.14 ± 0.14	1	-	-	-	-	-	-
*Halichoeresmelanurus* (Bleeker 1851)	6	0.85 ± 0.4	1	-	-	-	1	3	1
*Halichoerespodostigma* (Bleeker 1854)	6	0.85 ± 0.85	-	-	6	-	-	-	-
*Halichoeresrichmondi* (Fowler & Bean 1928)	3	0.42 ± 0.29	-	-	1	-	-	2	-
*Labroidesdimidiatus* (Valenciennes 1839)	20	2.85 ± 0.98	-	3	6	6	4	1	-
*Oxycheilinusorientalis* (Günther 1862)	3	0.42 ± 0.29	-	-	-	-	1	2	-
*Oxycheilinusunfasciatus* (Streets 1877)	1	0.14 ± 0.14	-	-	-	-	1	-	-
*Thalassomalunare* (Linnaeus 1758)	66	9.42 ± 3.19	-	-	5	20	9	20	12
Liopropomatidae									
*Diploprionbifasciatum* (Cuvier 1828)	2	0.28 ± 0.18	-	-	-	-	1	1	-
Lutjanidae									
*Caesiocuning* (Bloch 1791)	55	7.85 ± 6.34	-	45	10	-	-	-	-
*Caesioteres* (Seale 1906)	100	14.28 ± 7.51	-	-	-	50	30	-	20
*Lutjanusbiguttatus* (Valenciennes 1830)	1	0.14 ± 0.14	-	-	-	-	1	-	-
*Lutjanusdecussatus* (Cuvier 1828)	1	0.14 ± 0.14	-	-	-	1	-	-	-
Mullidae									
*Parupeneusmacronemus* (Lacepède 1801)	1	0.14 ± 0.14	-	-	1	-	-	-	-
*Upeneustragula* (Richardson 1846)	4	0.57 ± 0.42	-	-	-	-	-	1	3
Nemipteridae									
*Scolopsisbilineata* (Bloch 1793)	2	0.28 ± 0.28	-	-	-	2	-	-	-
*Scolopsismargaritifera* (Cuvier 1830)	8	1.14 ± 0.45	-	-	-	3	2	2	1
Plotosidae									
*Plotosuslineatus* (Thunberg 1787)	330	47.14 ± 31.37	-	130	-	-	-	200	-
Pomacanthidae									
*Chaetodontoplusmesoleucus* (Bloch 1787)	2	0.28 ± 0.28	-	-	-	-	2	-	-
Pomacentridae									
*Abudefdufvaigiensis* (Quoy & Gaimard 1825)	125	17.85 ± 8.16	11	52	43	19	-	-	-
*Amblyglyphidodonaureus* (Cuvier 1830)	3	0.42 ± 0.29	-	2	-	-	1	-	-
*Amblyglyphidodonleucogaster* (Bleeker 1847)	7	1 ± 0.84	-	-	-	-	6	1	-
*Amblypomacentrusbreviceps* (Schlegel & Müller 1840)	5	0.71 ± 0.71	5	-	-	-	-	-	-
*Amphiprionakallopisos* (Bleeker 1853)	2	0.28 ± 0.28	-	-	-	-	2	-	-
*Amphiprionfrenatus* (Brevoort 1856)	2	0.28 ± 0.28	-	-	2	-	-	-	-
*Chromisanalis* (Cuvier 1830)	1	0.14 ± 0.14	1	-	-	-	-	-	-
*Chromisnotata* (Temminck & Schlegel 1843)	331	47.28 ± 17.2	8	99	45	-	24	120	35
*Chrysipterabrownriggii* (Bennett 1828)	3	0.42 ± 0.42	-	-	-	3	-	-	-
*Chrysipteraglauca* (Cuvier 1830)	408	58.28 ± 42.93	-	-	3	15	312	22	56
*Chrysipterarollandi* (Whitley 1961)	20	2.85 ± 1.43	-	4	8	-	-	8	-
*Chrysipteraspringeri* (Allen & Lubbock 1976)	239	34.14 ± 22.66	4	-	-	150	85	-	-
*Neopomacentrusnemurus* (Bleeker 1857)	67	9.57 ± 8.46	-	-	7	-	60	-	-
*Pomacentrusamboinensis* (Bleeker 1868)	27	3.85 ± 2.49	18	-	4	-	-	5	-
*Pomacentrusauriventris* (Allen 1991)	4	0.57 ± 0.57	4	-	-	-	-	-	-
*Pomacentrusburroughi* (Fowler 1918)	161	23 ± 9.63	7	14	3	9	60	8	60
*Pomacentrusstigma* (Fowler & Bean 1928)	78	11.14 ± 5.09	-	26	-	7	-	12	33
Scaridae									
*Chlorurusbleekeri* (de Beaufort 1940)	18	2.57 ± 1.63	-	-	-	-	1	6	11
*Chlorurussordidus* (Forsskål 1775)	49	7 ± 2.5	-	11	-	-	11	13	14
*Scarusflavipectoralis* (Schultz 1958)	36	5.14 ± 3.26	-	-	-	12	2	-	22
*Scarusghobban* (Fabricius 1775)	3	0.42 ± 0.42	-	-	-	3	-	-	-
*Scarusglobiceps* (Valenciennes 1840)	61	8.71 ± 8.71	-	-	-	-	-	61	-
*Scarusrusselii* (Valenciennes 1840)	2	0.28 ± 0.18	-	-	-	1	1	-	-
Siganidae									
*Siganuscorallinus* (Valenciennes 1835)	2	0.28 ± 0.28	-	-	-	-	-	-	2
*Siganuspuellus* (Schlegel 1852)	4	0.57 ± 0.36	-	-	2	2	-	-	-
*Siganusvirgatus* (Valenciennes 1835)	2	0.28 ± 0.28	-	-	-	-	-	2	-
*Siganusvulpinus* (Schlegel & Müller 1845)	10	1.42 ± 0.52	-	-	-	3	2	3	2
Zanclidae									
*Zancluscornutus* (Linnaeus 1758)	1	0.14 ± 0.14	-	-	-	-	1	-	-

**Table 3.** General ecological indices at each site surveyed

Ecological Indices	Pulau Baik	Terumbu Misan-misan 1	Terumbu Misan-misan 2	Pulau Tabawan	Terumbu Batik	Terumbu Tingkayu	Terumbu Maganting
Shannon-Wiener Diversity (H')	2.04	1.91	2.12	2.16	1.83	1.95	2.34
Simpson Diversity (1-D)	0.84	0.8	0.81	0.79	0.72	0.76	0.87
Dominance Index (C)	0.16	0.2	0.19	0.21	0.28	0.24	0.13
Pielou's Evenness (E)	0.7	0.48	0.49	0.38	0.21	0.27	0.45

**Table 4.** Analysis of Similarity (ANOSIM) of priori groups, based on proximity to shore and reef type. Italicised value indicates significance, p < 0.05.

**ANOSIM (One-Way)**		
**Factors**	**R**	**p**
Proximity to shore	0.648	*0.029*
Reef type	0.111	0.314

**Table 5.** Results of SIMPER analysis of species contributing most to assemblage differences between clusters. Symbol “*” indicates species were not representative of the sites due to species low relative abundance as compared to other species at the particular sites.

**Species**	**Priori groups**						
	**Average abundance**				**Dissimilarities contribution (%)**		
	**Outlier A**	**Cluster B**	**Cluster C**		**A & B**	**A & C**	**B & C**
*Abudefdufvaigiensis*	3.32	4.59	1.45		5.62	3.18	4.58
*Aeoliscusstrigatus*	-	-	1.83		*	2.34	2.01
*Amblypomacentrusbreviceps*	2.24	-	-		3.62	2.8	*
*Caesiocuning*	-	3.29	-		5.62	*	3.68
*Caesioteres*	-	-	5.67		*	*	6.1
*Chlorurussordidus*	-	2.31	2.35		3.27	2.93	1.84
*Chromisnotata*	2.83	9.2	3.61		9.79	3.39	5.82
*Chrysipteraglauca*	-	2.14	9.67		3.16	11.51	8.03
*Chrysipterarollandi*	-	2.55	-		4.15	*	2.75
*Chrysipteraspringeri*	2	-	7.16		3.24	7.92	7.47
*Cirrhilabrusrubripinnis*	-	*	1.49		*	1.91	*
*Labroidesdimidiatus*	-	1.73	9.67		2.97	*	*
*Neopomacentrusnemurus*	-	0.88	2.58		*	2.8	2.95
*Plotosuslineatus*	-	8.51	-		11.99	*	8.44
*Pomacentrusamboinensis*	4.24	1.41	-		4.57	5.31	*
*Pomacentrusauriventris*	2	-	-		3.24	2.51	*
*Pomacentrusburroughi*	2.65	2.77	6.16		*	4.36	3.87
*Pomacentrusstigma*	-	2.85	2.8		4.14	3.79	2.82
*Scarusflavipectoralis*	-	-	3.19		*	4.16	3.55
*Scarusglobiceps*	-	2.6	-		3.34	*	2.43
*Thallasomalunare*	-	2.24	3.65		3.41	4.6	2.14
*Chlorurusbleekeri*	*	0.82	1.44		*	*	1.64

## References

[B7593218] Advani Sahir, Rix Laura N., Aherne Danielle M., Alwany Magdy A., Bailey David M. (2015). Distance from a fishing community explains fish abundance in a no-take zone with weak compliance. PLOS One.

[B7593927] Affendi YA, Ho N, Kee-Alfian AA, Aazani M, Muhammad Ali SH, Nara A, Munirah SS, Kassem KR, Hoeksema BW, Affendi YA (2012). Semporna marine ecological expedition.

[B7593283] Andersson K (2002). A study of coral reef fishes along a gradient of disturbance in the Langkawi Archipelago, Malaysia.

[B7593311] Araujo Gonzalo, Agustines Ariana, Tracey Brian, Snow Sally, Labaja Jessica, Ponzo Alessandro (2019). Photo-ID and telemetry highlight a global whale shark hotspot in Palawan, Philippines. Scientific Reports.

[B7593322] Bellwood David R., Hoey Andrew S., Hughes Terence P. (2011). Human activity selectively impacts the ecosystem roles of parrotfishes on coral reefs. Proceedings of the Royal Society B: Biological Sciences.

[B7595652] Bernama (2019). Sabah Parks seeks to gazette Lahad Datu's Darvel Bay by 2020.. https://www.malaysiakini.com/news/467761.

[B7595660] Bernama (2019). RM6 billion lost each year to illegal fishing. https://www.malaysiakini.com/news/490566.

[B7595668] Bernama (2019). Fish bomb: Police nab 10 men, inspect 95 fishing boats in Semporna. https://www.thesundaily.my/local/fish-bomb-police-nab-10-men-inspect-95-fishing-boats-in-semporna-YH1081001.

[B7595676] Bernama (2021). Sabah in process to gazette two marine parks. https://www.dailyexpress.com.my/news/168866/sabah-in-process-to-gazette-two-marine-parks/.

[B7595684] Bernama (2021). Lahad Datu MMEA arrest two fishermen over fish bombing activities. https://www.thestar.com.my/news/nation/2021/05/05/lahad-datu-mmea-arrest-two-fishermen-over-fish-bombing-activities.

[B7593705] Biusing R (2001). Assessment of coastal fisheries in the Malaysia-Sabah portion of the Sula-Sulawesi Maritime Ecoregion (SSME).

[B7593713] Burke L, Selig E, Spalding M (2002). Reefs at risk in Southeast Asia.

[B7593721] Cem PS, Assim Z (1996). Water quality of Gunung Silam watershed and adjoining fringes.

[B7667251] Chao A. (1984). Non-parametric estimation of the number of classes in a population. *Scandinavian Journal of Statistics*.

[B7667242] Chao Anne (1987). Estimating the Population Size for Capture-Recapture Data with Unequal Catchability. Biometrics.

[B7593847] Cielo Filho R., Martins F. R., Gneri A. (2002). Fitting abundance distribution models in tropical arboreal communities of SE Brazil. Community Ecology.

[B7666777] Clarke K. Robert, Somerfield Paul J., Gorley Raymond N. (2008). Testing of null hypotheses in exploratory community analyses: similarity profiles and biota-environment linkage. Journal of Experimental Marine Biology and Ecology.

[B7593730] Clark KR, Gorley RN (2015). Primer v.7: User manual/tutorial.

[B7667233] Colwell Robert K., Elsensohn Johanna E. (2014). EstimateS turns 20: statistical estimation of species richness and shared species from samples, with non-parametric extrapolation. Ecography.

[B7593751] Daw T (2004). Fish aggregations in Sabah, East Malaysia.

[B7593767] De Silva MWRN, Canbanban H, Ditlev H, Ridzwan AR, De Silva MWRN, Ridzwan AR, Saleem M, Cabanban AS (1999). Ekspedisi Galaxea’98. A study of living marine resources of Darvel Bay, Sabah, Malaysia..

[B7593788] Ditlev H, De Silva MWRN, Ridzwan AR, Toerring D, Widt S, De Silva MWRN, Ridzwan AR, Saleem M, Cabanban AS (1999). Ekspedisi Galaxea’98. A study of living marine resources of Darvel Bay, Sabah, Malaysia.

[B7593812] Ditlev H (2003). New scleractinian corals (Cnidaria: Anthozoa) from Sabah, North Borneo. Description of one new genus and eight new species, with notes on their taxonomy and ecology. Zoologische Mededelingen.

[B7593830] Dorman Stacey R., Harvey Euan S., Newman Stephen J. (2012). Bait effects in sampling coral reef fish assemblages with Stereo-BRUVs. PLOS One.

[B7593839] Farhana-Azmi N (2019). Personal observation.

[B7667224] Foggo A, Attrill MJ, Frost MT, Rowden AA (2003). Estimating marine species richness: an evaluation of six extrapolative techniques. Marine Ecology Progress Series.

[B7659922] Fricke R., Eschmeyer W. N, Van der Laan R. Eschmeyer's Catalog of Fishes. https://researcharchive.calacademy.org/research/ichthyology/catalog/fishcatmain.asp.

[B7593872] Gordon Arnold, Sprintall Janet, Ffield Amy (2011). Regional oceanography of the Philippine Archipelago. Oceanography.

[B7667132] Graham N. A. J., Wilson S. K., Jennings S., Polunin N. V. C., Bijoux J. P., Robinson J. (2006). Dynamic fragility of oceanic coral reef ecosystems. Proceedings of the National Academy of Sciences.

[B7593881] Hammer O, Harper DAT, Ryan PD (2001). PAST: paleontological statistics software for education and data analysis. Palaentologia Electronica.

[B7593899] Harvey ES, McLean DL, Frusher S, Haywood MDD, Newman SJ, Williams A (2013). The use of BRUVS as a tool for assessing marine fisheries and ecosystems: A review of the hurdles and potential.

[B7593909] Hill Jane K., Hamer Keith C. (2004). Using species abundance models as indicators of habitat disturbance in tropical forests. Journal of Applied Ecology.

[B7593953] Hoeksema BW, Suharsono, Cleary DFR, Hoeksema BW (2004). Marine biodiversity of the coastal area of the Berau region, East Kalimantan, Indonesia. Progress report East Kalimantan Program - Pilot phase (October 2003): Preliminary results of a field survey performed by an Indonesian-Dutch biodiversity research team.

[B7593967] Hoeksema BW, Renema W (2007). Biogeography, time and place: Distributions, barriers and islands.

[B7593980] Hoeksema BW, Hoeksema BW, Van der Meij SET (2008). Cryptic marine biota of the Raja Ampat Islands group. Progress report Ekspedisi Widya Nusantara.

[B7593918] Ho N, Kassem KR (2009). Reef status of Semporna Priority Conservation Area.

[B7667123] Jones G. P., McCormick M. I., Srinivasan M., Eagle J. V. (2004). Coral decline threatens fish biodiversity in marine reserves. Proceedings of the National Academy of Sciences.

[B7593993] Khalaf MA, Kochzius M (2002). Changes in trophic community structure of shore fishes at an industrial site in the Gulf of Aqaba, Red Sea. Marine Ecology Progress Series.

[B7594002] Lee W, Chou LM (2003). The status of coral reefs of Pulau Banggi and its vincinity, Sabah, based on surveys in June 2003. https://www.researchgate.net/publication/309131272_The_status_of_coral_reefs_of_Pulau_Banggi_and_its_vicinity_Sabah_based_on_surveys_in_June_2003.

[B7594010] Longenecker K, Langston R, Bolick H, Kondio U (2011). Reproduction, catch, and size structure of exploited reef-fishes at Kamiali Wildlife Management Area, Papua New Guinea. http://hbs.bishopmuseum.org/publications/pdf/tr57.pdf.

[B7594018] Magurran AE (2004). Measuring biological diversity..

[B7595692] Miwil O (2021). Fishermen nabbed for using explosives to catch fish off Lahad Datu.. https://www.nst.com.my/news/nation/2021/05/688037/fishermen-nabbed-using-explosives-catch-fish-lahad-datu.

[B7594026] Norhadi I (1996). Benthic marine plants off Mt. Silam, Darvel Bay, Sabah.

[B7594034] Nyström Magnus, Folke Carl, Moberg Fredrik (2000). Coral reef disturbance and resilience in a human-dominated environment. Trends in Ecology & Evolution.

[B7595404] Othman MA, Lee (1999). Coastal zone management necessity in Malaysia.

[B7595429] Pilcher NJ (1996). Coral reef fish population structure and distribution of Mt. Silam, Darvel Bay, Sabah.

[B7595421] Pilcher NJ, Cabanban A (2000). The status of coral reefs in Sabah, Labuan and Sarawak, East Malaysia..

[B7595700] Ralon L (2019). Sabah upbeats 13 percent marine protection. https://www.dailyexpress.com.my/news/132423/sabah-parks-upbeat-on-13pc-marine-protection-/.

[B7595453] Malaysia Reef Check (2017). Status of coral reefs in Malaysia 2017. https://www.reefcheck.org.my/reports.

[B7595445] Reef Check Malaysia (2013). Status of coral reefs in Malaysia 2013. https://www.reefcheck.org.my/reports.

[B7595461] Reef Check Malaysia (2019). Status of coral reefs in Malaysia, 2019. https://www.reefcheck.org.my/reports.

[B7667144] Renfro Bobbie, Chadwick Nanette E. (2017). Benthic community structure on coral reefs exposed to intensive recreational snorkeling. PLOS One.

[B7595502] Saleh E, Hoque MA, Rahman RA (2007). Water circulation in Darvel Bay, Sabah, Malaysia. OCEANS 2007 – Europe.

[B7595478] Sale PF (1993). The ecology of fishes on coral reefs.

[B7595511] Santodomingo Nadiezhda, Perry Chris, Waheed Zarinah, Syed Hussein Muhammad Ali bin, Rosedy Allia, Johnson Kenneth G. (2021). Marine litter pollution on coral reefs of Darvel Bay (East Sabah, Malaysia). Marine Pollution Bulletin.

[B7595522] Stallings Christopher D. (2009). Fishery-independent data reveal negative effect of human population density on Caribbean predatory fish communities. PLOS One.

[B7595717] Toyos L (2019). Fish bombing, illegal fishing arrests in Semporna and Tawau. http://www.dailyexpress.com.my/news/132637/fish-bombing-illegal-fishing-arrests-in-semporna-and-tawau/.

[B7667260] Waheed Z., Hoeksema B. W. (2012). A tale of two winds: species richness patterns of reef corals around the Semporna peninsula, Malaysia. Marine Biodiversity.

[B7595558] Walsh Rory P. D. (2009). Drought frequency changes in Sabah and adjacent parts of northern Borneo since the late nineteenth century and possible implications for tropical rain forest dynamics. Journal of Tropical Ecology.

[B7595567] Whittaker R. H. (2019). Evoluntion and measurement of species diversity. Taxon.

[B7595576] Wilson S. K., Fisher R., Pratchett M. S., Graham N. A. J., Dulvy N. K., Turner R. A., Cakacaka A., Polunin N. V. C. (2010). Habitat degradation and fishing effects on the size structure of coral reef fish communities. Ecological Applications.

[B7595598] Wood EM, Dipper F, Angkaji A (2004). Patterns of change in reef communities revealed by the monitoring programme for the Semporna Island reefs, Sabah, Malaysia. Proceedings of 10th International Coral Reef Symposium.

[B7595626] Wood EM, Ng JV (2016). Acoustic detection of fish bombing. https://lighthouse-foundation.org/Binaries/Binary1075/50211-mcs-Acoustic-detection-of-fish-bombing-final-report-01-2016.pdf.

[B7595634] Wraith J, Lynch T, Minchinton TE, Broad A, Davis AR (2013). Bait type affects fish assemblages and feeding guilds observed at baited remote underwater video stations. Marine Ecology Progress Series.

